# A Cytosolic Sensor, *Pm*DDX41, Binds Double Stranded-DNA and Triggers the Activation of an Innate Antiviral Response in the Shrimp *Penaeus monodon* via the STING-Dependent Signaling Pathway

**DOI:** 10.3389/fimmu.2019.02069

**Published:** 2019-08-29

**Authors:** Suthinee Soponpong, Piti Amparyup, Taro Kawai, Anchalee Tassanakajon

**Affiliations:** ^1^Department of Biochemistry, Faculty of Science, Center of Excellence for Molecular Biology and Genomics of Shrimp, Chulalongkorn University, Bangkok, Thailand; ^2^National Center for Genetic Engineering and Biotechnology (BIOTEC), National Science and Technology Development Agency, Pathumthani, Thailand; ^3^Laboratory of Molecular Immunobiology, Division of Biological Science, Graduate School of Science and Technology, Nara Institute of Science and Technology, Nara, Japan

**Keywords:** antiviral immune response, DDX41, immune signaling pathway, *Penaeus monodon*, STING

## Abstract

Helicase DDX41 is a cytosolic sensor capable of detecting double-stranded DNA in mammals. However, the function of DDX41 remains poorly understood in invertebrates. In a previous study, we identified the first DDX41 sensor in the black tiger shrimp *Penaeus monodon* (*Pm*DDX41) and showed that it played a role in anti-viral response. In the present study, we demonstrated that *PmDDX41* was localized in the cytoplasm of shrimp hemocytes. However, *PmDDX41* was localized in both the cytoplasm and nucleus of hemocytes in the presence of white spot syndrome virus (WSSV) infection or when stimulated by the nucleic acid mimics, poly(dA:dT) and poly(I:C). Similar results were observed when *Pm*DDX41 was transfected into human embryonic kidney 293T (HEK293T) cells. Immunoprecipitation further demonstrated that *Pm*DDX41 bound to biotin-labeled poly(dA:dT) but not poly(I:C). The overexpression of shrimp *Pm*DDX41 and mouse stimulator of interferon gene (*Mm*STING) in HEK293T cells synergistically promoted IFN-β and NF-κB promoter activity via the DEADc domain. Co-immunoprecipitation (Co-IP) also confirmed that there was an interaction between *Pm*DDX41 and STING after stimulation with poly(dA:dT) but not poly(I:C). Our study is the first to demonstrate that *Pm*DDX41 acts as a cytosolic DNA sensor that interacts with STING via its DEADc domain to trigger the IFN-β and NF-κB signaling pathways, thus activating antiviral innate immune responses.

## Introduction

The recognition of pattern recognition receptors (PRRs)-mediated pathogen-associated molecule patterns (PAMPs) is the first line of a host's innate immune response to pathogenic infection (1). The innate immune recognition mechanisms of invertebrates and vertebrates are perceived to share similar elements. Innate immune DNA/RNA sensing pathways detect foreign nucleic acids (bacterial or viral RNA and DNA) via PRRs to eliminate pathogenic infection and do so by initiating downstream signaling cascades that release inflammatory cytokines and interferons (IFNs); these subsequently limit viral replication and clearance of virus-infected cells (2, 3). In mammals, nucleic acid recognitions have been found such as toll-like receptors (TLRs), retinoic acid-inducible gene I (RIG-I)-like receptors (RLRs) and the group of cytosolic DNA sensor molecules, including DEAD (Asp-Glu-Ala-Asp) box polypeptide 41 (DDX41) and cyclic GMP-AMP synthase (cGAS). Of these receptors, DDX41 is a newly cytosolic DNA molecule which plays a role in a variety of innate immune response and is associated with several vertebrate diseases, including acute myeloid leukemia and myelodysplastic syndrome (4).

DDX41, an intracellular DNA, is a part of the DEAD-box protein group. Previous work in mice showed that DDX41 was a crucial cytosolic DNA sensor and was mediated by the adaptor stimulator of interferon gene (STING) in a mouse DC line (D2SC cell line) (5). DDX41 composes of two RecA-like domains, DEADc, HELICc and zinc finger, which are known to be involved in ATP binding, RNA unwinding and nucleic acid recognition (6). Functionally, DDX41 binds directly to dsDNA and STING protein via its DEADc domain and subsequently triggers the activation of NF-κB to produce IFN-β in myeloid dendritic cells (5). DEADc domain of DDX41 has also been shown to associate with STING leading to activate TANK-binding kinase 1 (TBK1) and trigger the production of proinflammatory cytokines and IFNs (7). Research has also demonstrated that dsRNA/DNA can activate STING to trigger the downstream molecule; signal transduction and activator of transcription 6 (STAT6) and then produce the expression of specific genes such as CCL2 and CCL20 which recruit immune cells to defense against viral infection (8). Consequently, it is possible that DDX41 acts as the upstream sensor for this STING-STAT6-mediated signaling pathway (9). Moreover, a recent study isolated a DDX41 homolog from the orange spotted grouper *Epinephelus coioides* (*Ec*DDX41) and showed that this homolog regulated mitochondrial antiviral-signaling protein (MAVS) and TBK1-induced IFN immune response (10).

Thus far, our knowledge of DDX41 has mainly originated from vertebrates such as the human and mouse. In order to fully characterize the biological function of DDX41 involved in the innate immunity, a range of different model organisms has been developed, particularly lower vertebrates, which possess a well-established and complicated innate immune system (11). In *Drosophila*, Abstrakt acts as a homolog of DDX41, was previously reported to play important roles for survival at all stages throughout the life cycle (12). However, Abstrakt has not been studied with regards to antiviral immune response and little is known about its occurrence and existence in other organisms, particularly in invertebrates. More recent studies reported that *Dm*STING was transcriptionally induced by Zika virus (ZIKV) infection and plays an anti-ZIKV role in the *Drosophila* brain and activated the immune deficiency (IMD) pathway, which functions as the inflammatory NF-κB pathway in this insect (13, 14)*. Cg*STING was identified from the complete genome of the Pacific oyster *Crassostrea gigas* and shown to be induced by Ostreid herpesvirus-1 (OsHV-1) inoculation and poly(I:C) stimulation (15). *Cg*STING binds to cyclic dinucleotides (CDNs) and interacts with TBK1 (16). However, little is known about how the STING-TBK1-IRFs/NF-κB signaling pathway is activated, or how antiviral effectors are produced.

DDX41 has also been identified in the black tiger shrimp *Penaeus monodon* (*Pm*DDX41); the expression of *Pm*DDX41 was up-regulated in response to white spot syndrome virus (WSSV), a DNA virus with severe pathogenic effects on shrimp populations. Silencing the *Pm*DDX41 gene resulted in the rapid death of WSSV-infected shrimp and a reduction in the expression levels of several immune genes related to immune signaling pathways and antimicrobial peptides (17). In this study, we further characterized the function of *Pm*DDX41 as a nucleic acid sensor in shrimp and investigated how this sensor interacts with dsDNA and STING to send signals that ultimately activated innate immune responses in a human embryonic kidney cell line (HEK293T). Our data provide evidence that *Pm*DDX41 acts as a cytosolic sensor that binds directly to DNA and triggers the STING-mediated signaling pathway, thus activating the host anti-viral immune response.

## Materials and Methods

### Experimental Shrimp

Healthy black tiger shrimp *P. monodon* were kindly provided by Charoen Pokphand Foods in Chanthaburi province, Thailand with average body mass of 10–15 g. The shrimp were maintained in aerated seawater (20‰) at an ambient temperature of about 28 ± 1°C for 1 week prior to the experiment. To determine the expression level of the *Pm*DDX41 transcript, we collected hemocytes from triplicate groups of shrimp (*n* = 3 for each group) as described previously (18). All samples were then stored at −80°C to await RNA extraction. This study was conducted under the ethical principles and guidelines according to the animal use protocol approved by Chulalongkorn university Animal Care And USE Committee (CU-ACUC).

### Cells and Reagents

HEK293T cells (CH3 BioSystems) were cultured in Dulbecco's modified Eagle's medium (DMEM) (Nacalai Tesque) containing 10% heat-inactivated fetal bovine serum (FBS) (Life Technologies) in a 5% CO_2_ incubator. HMW polyinosinic-polycytidylic acid (poly(I:C)) (InvivoGen) and polydeoxyadenylic-deoxythymidylic acid sodium salt (poly(dA:dT)) (InvivoGen) were prepared in accordance with the manufacturer's protocol and mixed with Lipofectamine 2000 (Life Technologist) at a ratio of 1:1 (μg/μl) in Opti-MEM (Life Technologies) for cell stimulation. The following antibodies were used: anti-DDX41 (Abcam), anti-Flag (Sigma), and anti-Myc (Sigma).

### Total RNA Extraction and Reverse Transcription

Hemocytes were first homogenized in GENEzol (Geneaid) and then total RNA was extracted in accordance with the manufacturer's protocol. Prior to downstream application, total RNA was treated with DNaseI (NEB). One microgram RNA was reverse transcribed in single-stranded cDNA synthesis using RevertAid First Strand cDNA Synthesis Kit (Thermo Fisher Scientific). Synthesized cDNA was stored at −20°C until use.

### Localization of *Pm*DDX41 in Hemocytes Following Stimulation With WSSV and Nucleic Acid Mimics

In order to investigate the localization of *Pm*DDX41 protein in shrimp hemocytes, we injected five shrimp (15 ± 1 g individual body weight) with 50 μl of WSSV (10^5^ copies/ml), poly(dA:dT) (2 μg/g per shrimp) and high molecular weight (HMW) poly(I:C) (2 μg/g per shrimp) in PBS buffer for 48 h. Phosphate buffered saline (PBS; 137 mM NaCl, 2.7 mM KCl, 4.3 mM Na_2_HPO_4_, 1.4 mM KH_2_PO_4_) buffer was injected into another group of shrimp as a control. Subsequently, hemolymph was drawn and fixed in 4% paraformaldehyde (at a ratio of 1:1) for 10 min at room temperature. Then, hemocytes were separated by centrifugation and suspended with 1X PBS (pH 7.4), counted by hemocytometer. Hemocytes were then mounted onto poly-L-lysine slide (Thermo Scientific) at a density of 1 × 10^6^ cells/slide. The cells were then washed three times in 1X PBS (pH 7.4), permeabilized by 1% tritonX-100 in 1X PBS (pH 7.4) for 5 min at room temperature, and finally washed three times with 1X PBS (pH 7.4) for 5 min. Next, the cells were blocked with 10% fetal bovine serum (FBS) in 1X PBS (pH7.4) at room temperature for 1 h. Cells were then probed with a 1:1,000 dilution of purified rabbit polyclonal anti-human-DDX41 antibody (Abcam) at room temperature for 3 h; negative controls were incubated with 1% FBS in 1X PBS (pH7.4). Subsequently, the slides were washed and probed with a 1:1,000 dilution of goat anti-rabbit antibody conjugated with Alexa Fluor 568 in 1% FBS in 1X PBS (pH 7.4) at room temperature for 1 h. To stain nuclear DNA, the cells were probed with Hoechst 33342 (blue) before mounting with medium Prolong? Gold antifade reagent. Finally, fluorescence images were detected by LSM 700 laser scanning confocal microscope (Carl Zeiss).

### Gene Expression Profiles in Response to the Injection of Nucleic Acid Mimics

To measure the changes in *Pm*DDX41 transcript levels using quantitative RT-PCR (qRT-PCR) in *P. monodon* after injection with the nucleic acid mimics poly(dA:dT) and poly(I:C). For nucleic acid injection, shrimp (10 ± 1 g in individual body weight) were divided into triplicate groups of three shrimp per group and injected with 50 μl of poly(dA:dT) (2 μg/g) diluted in PBS and 50 μl of HMW poly(I:C) (2 μg/g) diluted in PBS in the second abdominal segment (50 μL per shrimp). The control group was injected with PBS (pH 7.4). The experimental shrimp were reared in seawater tanks, and hemocyte cell pellets were randomly collected at 0, 6, 12, 24, and 48 h post-injection (hpi) for RNA extraction. Total RNA extraction and first-strand cDNA synthesis were performed following the methods described above. The extracted total RNAs from three shrimp per treatment at each time point were then pooled. qRT-PCR analysis was then performed as described by Amparyup et al. (18) using *Pm*DDX41-F and *Pm*DDX41-R primers (Table 1). EF1-α was amplified as an internal control and reference standard to verify the qRT-PCR reaction. Three replicates were performed for each template, and three independent replicates were utilized for each data point. Ct values for the infected hemocyte samples at each time point were normalized using the saline-injected samples. The relative expression ratio was determined by the mathematical model described by Pfaffl (19).

**Table 1:** List of nucleotide primer used in the experiments.

**Primer name**	**Sequence (5^**′**^ to 3^**′**^)**	**Purpose**
*Pm*DDX41-F	AGCCCTTCAAGGACGTGACATGA	Transcription study
*Pm*DDX41-R	GCATATCTATGAGGCGTCCTGGA	
Myc_*Pm*DDX41_BamHI-F	AAGGATCCAAAGATGGACAGCCCGAAGAAGCTC	Protein expression in HEK293T cell
Myc_*Pm*DDX41_XhoI-R	AAGCTCGAGGTAATCAGCTGCATTAGCAGCCAA	
Myc_Dead*Pm*DDX41_XhoI-R	AAGCTCGAGAGCAAAGATAAGTACTGGTGGAG	
Myc_Helic*Pm*DDX41_BamHI_F	AAGGATCCAAAGGATCTCATTCAGGAGTATTTG	
Flag_*Pm*DDX41_SalI_F	CGCGTCGACGTCGGCATGGACAGCCCGAAGAAGCTC	
Flag_*Pm*DDX41_BamHI_R	CGCGGATCCGCGTTAGTAATCAGCTGCATTAGCAGCCAA	
Flag_Dead*Pm*DDX41_BamHI_R	CGCGGATCCGCGTTAAGCAAAGATAAGTACTGGTGGAG	
Flag_Helic*Pm*DDX41_SalI_F	CGCGTCGACGTCGGCGATCTCATTCAGGAGTATTTG	

### Plasmid Construction

The full-length sequence of *Pm*DDX41 cDNA was cloned into pFlag-CMV5 (Sigma-Aldrich) and pcDNA3-Myc (Santa Cruz Biotechnology) expression vectors. A series of deletion mutations in the DEADc domain and HELICc domain of *Pm*DDX41 were then generated by PCR from *Pm*DDX41 cDNA (Table 1). Reporter plasmids for IFN-β and NF-κB, and expression vectors for IPS-1 and *Mm*STING, were then constructed as described previously (20, 21).

### Interactions Between *Pm*DDX41 Protein and Nucleic Acid Mimics

To investigate whether *Pm*DDX41 binds directly to DNA, we transfected HEK293T cells (1 × 10^6^ cell/ml) with 4 μg of *Pm*DDX41-Myc recombinant plasmid containing Myc tags using Lipofectamine 2000. After 24 h, cells were lysed by homogenization buffer (homo buffer; 150 mM NaCl, 5 mM EDTA pH 8.0, 25 mM Tris-HCl pH8.0, and 0.2% TritonX-100) and sonicated. The crude protein was then harvested by centrifugation at 13,000 rpm for 15 min and was then stored at −20° C. The nucleic acid mimic, poly (dA:dT), was then labeled with biotin using a Biotin DecaLabel DNA Labeling Kit (Thermo Scientific) in accordance with the manufacturer's instructions. In brief, 1 ng of poly(dA:dT) was mixed with 10 μl of 5X reaction buffer decanucleotide and adjusted with nuclease free-water to 44 μl. The contents of the tube were boiled for 5–10 min and then cooled on ice. Next, 5 μl of biotin labeling mix and 5 units of Klenow fragment exonuclease was added. The tube was then incubated at 37° C for 20 h and the reaction terminated by the addition of 1 μl of 0.5 M EDTA (pH 8.0). The labeled DNA was then stored at −20° C. The *Pm*DDX41 protein was immunoprecipitated with 500 ng of poly(dA:dT) and HMW poly(I:C)-labeled-biotin (InvivoGen) and a 1:250 dilution of anti-Myc-rabbit antibody overnight at 4° C. We then added protein A sepharose beads (GE) for 4 h at 4° C. Beads, bound to immunoprecipitates, were then washed 3 times with PBS buffer. Finally, whole-protein and immunoprecipitates were immunoblotted with the indicated antibodies.

### Luciferase Reporter Assay

HEK293T cells (1 × 10^5^ cell/ml) were plated into 24-well plates and transfected with 100 ng of reporter plasmid for IFN-β and NF-κB and 500 ng of expression plasmid or empty plasmid. As an internal control, 10 ng of pRL-TK (Promega) was used for transfection. The medium was replaced at 6 h post-transfection. After 24 h of transfection, cells were stimulated with 1 μg/ml of poly(dA:dT) and HMW poly(I:C). After 6 h stimulation, we measured the luciferase activity of HEK293T cells using a TriStar^2^ LB 942 Multi-detection Microplate Reader (Berthold) using the Dual-Glo Luciferase System (Promega) in accordance with the manufacturer's instructions.

### Immunofluorescence and Confocal Microscopy

Cells were cultured on poly-L-Lysine-coated coverslips in 24-well plates for 6 h and transfected with 1 μg of expression plasmid for 16 h. Cells were then stimulated with 1 μg/ml of poly(dA:dT) and HMW poly(I:C) for 6 h and fixed with 4% paraformaldehyde for 30 min. Cells were then washed three times in 0.02% TritonX-100 in PBS and permeabilized with PBS containing 100 mM glycine and 0.02% TritonX-100 for 30 min. Cells were then blocked in PBS with 10% FBS and 0.02% TritonX-100 at 4° C overnight and probed with a 1:100 dilution of primary antibody (anti-DDX41, anti-Flag, and/or anti-Myc) at 4° C overnight. The coverslips were then washed and incubated with secondary antibody (anti-mouse and/or anti-rabbit conjugated to Alexa Fluor 488 (InvitroGen) or 568 (InvitroGen)) at room temperature for 1 h. Nuclei were then probed with Hoechst 33342 (InvitroGen). Stained cells were finally mounted with Fluoro-KEEPER Antifade Reagent (Nacalai Tesque) and fluorescence images were acquired using an LSM 700 fluorescent microscope (Carl Zeiss).

### Expression of Recombinant *Pm*DDX41 and *Mm*STING Protein in HEK293T Cells

HEK293T cells (1 × 10^6^ cell/ml) were seeded into 10-cm dishes and transfected with 4 μg of *Pm*DDX41-Flag-tagged or *Mm*STING-Myc-tagged for 24 h. Cells were lysed with homo buffer and sonicated. After centrifugation of samples at 12,000 rpm at 4° C for 15 min, the recombinant proteins were collected. *Pm*DDX41 and *Mm*STING protein were analyzed by SDS-PAGE and detected by anti-Flag-tagged and anti-Myc-tagged antibodies.

### Co-immunoprecipitation

HEK293T cells (1 × 10^6^ cell/ml) were seeded into 10-cm dishes and transfected by Lipofectamine 2000 with 4 μg of expression plasmid encoding Flag and Myc tags. After 24 h, cells were stimulated with 1 μg/ml of poly(dA:dT) and HMW poly(I:C) for 6 h. Cells were then lysed with homo buffer containing protease inhibitor cocktail (Roche). After sonication, cell lysates were immunoprecipitated with a 1:500 dilution of anti-Myc-mouse antibody overnight at 4° C and then treated with protein A sepharose beads (GE) for 4 h at 4° C. Beads bound to immunoprecipitates were then washed three times with PBS buffer. Whole-cell lysates and immunoprecipitates were finally immunoblotted with the indicated antibodies.

### Western Blotting

HEK293T cells were cultured in six-well plates and lysed in lysis buffer (150 mM NaCl, 50 mM Tris-HCl pH 8, 0.5% Deoxycholate, 1% NP40, and 0.1% SDS). Following centrifugation, the supernatants were mixed with SDS sample buffer, separated by SDS-PAGE and transferred to an Immun-Blot PVDF membrane. Immunoblotting was then carried out with anti-DDX41 (Abcam), anti–Flag (Sigma), and anti–Myc (Sigma) antibodies. Bound antibodies were visualized with HRP-conjugated antibodies against mouse, rabbit, or goat IgG (Sigma-Aldrich) using Western Lighting Plus-ECL (PerkinElmer). HRP activity was detected using a LAS 4000 (Fujitsu Life Sciences).

## Results

### The Subcellular Localization of *Pm*DDX41 in *P. monodon* Hemocytes

**Figure 1:**
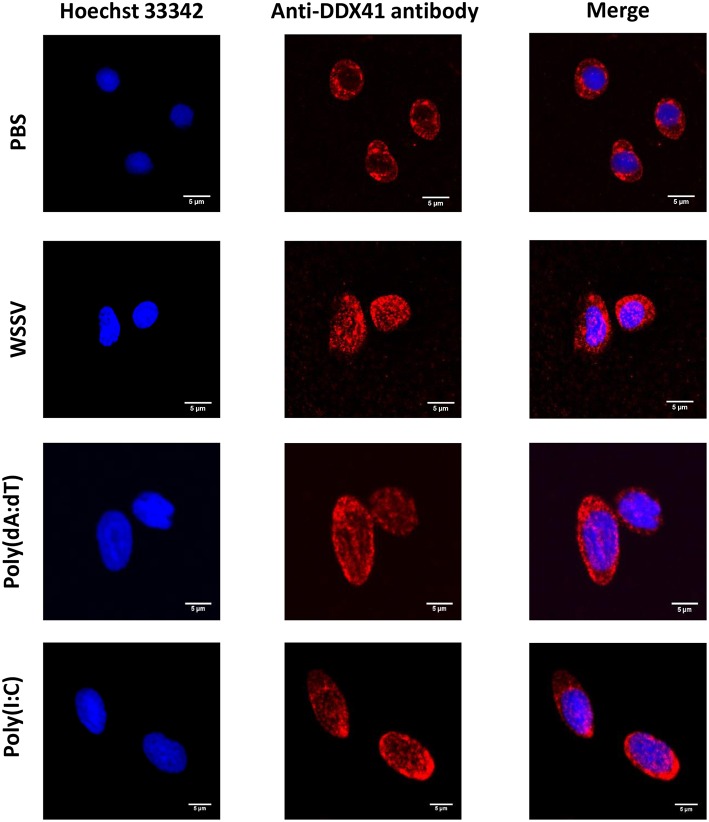
Subcellular localization of *Pm*DDX41 in shrimp hemocytes injected with PBS, WSSV, poly(dA:dT), or HMW poly(I:C), as visualized by fluorescence microscopy. Hemocyte cells were collected at 48 h post-injection and fixed with 4% paraformaldehyde. Hemocytes were then stained with anti-human-DDX41 antibody conjugated with Alexa Fluor 568 (red); nuclei were stained with Hoechst 33342 (blue). Scale bars represent 5 μm. Images were captured by laser-scanning confocal microscopy (Zeiss LSM-700; original magnification, 63×).

To explore the subcellular localization of *Pm*DDX41, hemocytes were collected from PBS-injected and WSSV-injected groups of shrimp and fixed with 4% paraformaldehyde. Hemocytes were then incubated with anti-DDX41 antibody followed by immunofluorescence detection. As shown in Figure 1, *Pm*DDX41 was mainly distributed in the cytoplasm of hemocytes from the PBS-injected shrimp. However, hemocytes from the WSSV-injected group showed increased levels of *Pm*DDX41 distributed in both the cytoplasm and nucleus. This indicated that *Pm*DDX41 was a cytosolic protein that translocated into the nucleus upon WSSV infection to mediate an antiviral immune response.

### Temporal Expression Profiles of the *Pm*DDX41 Transcript in *P. monodon* Hemocytes Following the Injection of Nucleic Acid Mimics

**Figure 2:**
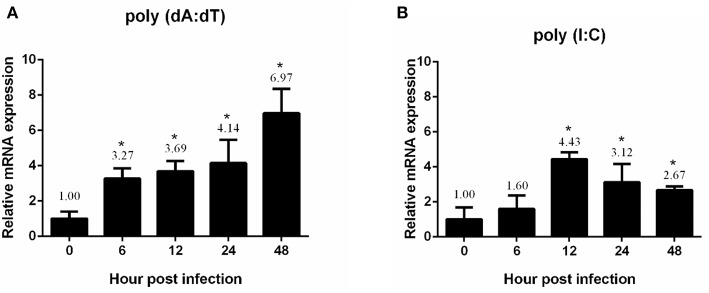
Expression profiles of *Pm*DDX41 in *P. monodon* hemocytes after injection of the nucleic acid mimics, poly(dA:dT) **(A)** and poly(I:C) **(B)**. Real time RT-PCR analysis of *Pm*DDX41 was performed in triplicate for each sample using the EF1-α gene as an internal control for normalization. Relative expression levels were calculated according to the method described by Pfaffl (19); data are shown as means ± SD of triplicate assays. The expression level at 0 h was set as a baseline (1.0). The asterisks above each bar indicate mean values that are significantly different (*p* < 0.05).

In a previous study, we showed that white spot syndrome virus (WSSV) (a double-stranded DNA virus) and yellow head virus (YHV) (a single-stranded RNA virus) could induce the expression of *PmDDX41* (17). Here, we injected the nucleic acid mimics, poly(dA:dT) and poly(I:C), and used qRT-PCR to investigate the effect of these injections on *Pm*DDX41 gene expression in shrimp hemocytes. Following the injection of poly(dA:dT), the expression of *Pm*DDX41 was significantly (*p* < 0.05) up-regulated at 6, 12, 24, and 48 h post injection; the highest levels (an increase of 6.97-fold) were observed 48 h after injection (Figure 2A). The injection of HMW poly(I:C) also induced a significant increase (*p* < 0.05) in *Pm*DDX41 expression at 12, 24, and 48 h by 4.43-, 3.12-, and 2.67-fold, respectively (Figure 2B).

### Localization of *Pm*DDX41 in HEK293T Cells

**Figure 3:**
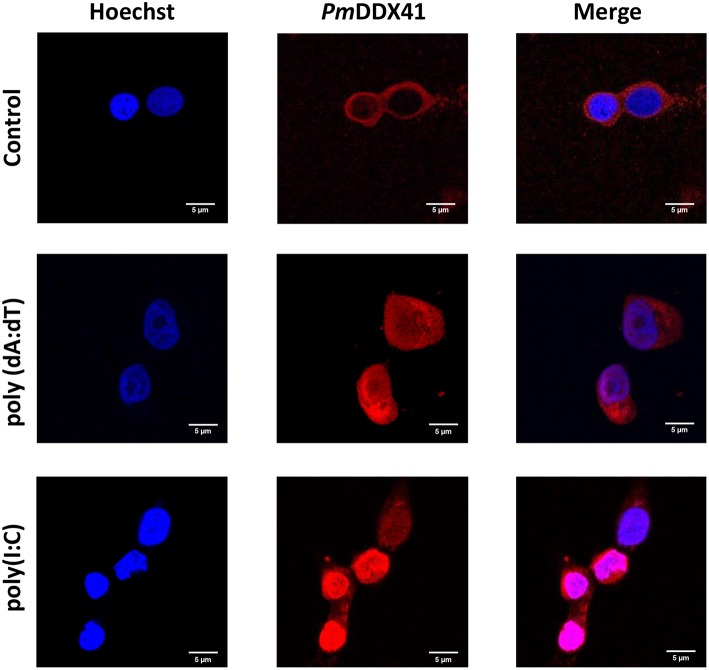
Localization of *Pm*DDX41 in HEK293T cells. Cells were transfected with a Myc-tagged-*Pm*DDX41 expression vector. After 24 h, cells were stimulated with 1 μg/ml of poly(dA:dT) and HMW poly(I:C) for 6 h. HEK293T cells were then stained with an anti-Myc antibody conjugated with Alexa Fluor 568 (red); nuclei were stained with Hoechst 33342 (blue). Scale bars represent 5 μm. Images were captured by laser-scanning confocal microscopy (Zeiss LSM-700; original magnification, 63×).

DDX41 has been reported to be highly conserved across a number of different species (17). We chose to use HEK 293T cells to further investigate the mechanism by which *Pm*DDX41 can sense nucleic acids. These cells are widely used as a model cell line for the mammalian cellular system. In this study, we used HEK293T cells as our model due to the lack of a stable crustacean cell line. First, we detected the localization of *Pm*DDX41 in HEK293T cells after transfection with a Myc-tagged-*Pm*DDX41 expression plasmid and staining with an anti-Myc antibody. We found that *Pm*DDX41 localized exclusively in the cytoplasm (Figure 3). Following stimulation by poly(dA:dT) and HMW poly(I:C) transfection, *Pm*DDX41 was observed in both the cytoplasm and nucleus (Figure 3); these findings were similar to those observed in shrimp hemocytes. These results confirmed that *Pm*DDX41 localized specifically to the cytoplasm in normal conditions but was induced and translocated into the nucleus following stimulation.

### Interactions Between *Pm*DDX41 and Nucleic Acid Mimics

**Figure 4:**
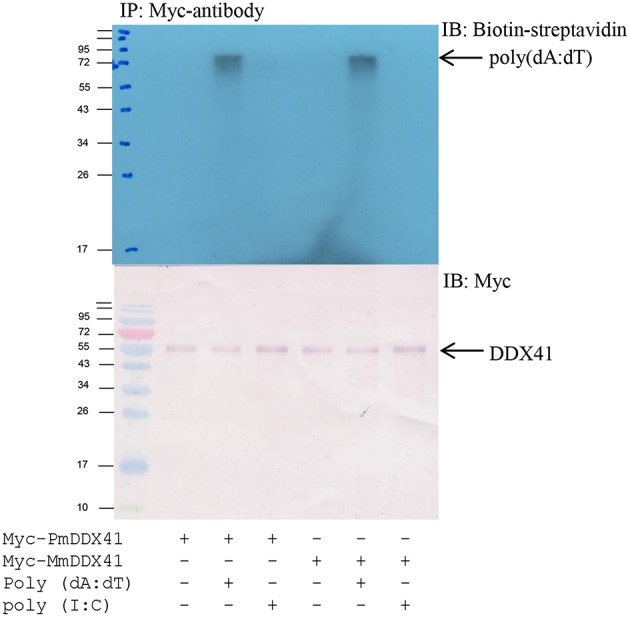
Interaction of *Pm*DDX41 with nucleic acid mimics. HEK293T cells were transfected with a Myc-tagged-*Pm*DDX41 expression vector for 24 h. Crude Myc-tagged-*Pm*DDX41 proteins were extracted and incubated with 500 ng of biotinylated poly(dA:dT) and HMW poly(I:C). Proteins were then immunoprecipitated with an anti-Myc antibody and analyzed by Western blotting using biotin-streptavidin detection and an anti-Myc antibody.

To determine whether *Pm*DDX41 binds directly to DNA, we transfected HEK293T cells with a plasmid encoding Myc-tagged *Pm*DDX41 recombinant protein. We used a Myc-tagged *Mm*DDX41 recombinant protein from the mouse as a control. The recombinant protein was incubated with biotin-labeled poly(dA:dT) and HMW poly(I:C), followed by the addition of agarose beads linked to an anti-Myc antibody. Only the recombinant DDX41 was immunoprecipitated by agarose beads plus poly(dA:dT) but not HMW poly(I:C) (Figure 4), thus indicating that *Pm*DDX41 is a DNA sensor molecule.

### The Overexpression of *Pm*DDX41 and *Mm*STING and Subsequent Effects on the Activation of IFN-β and NF-κB Promoters

**Figure 5:**
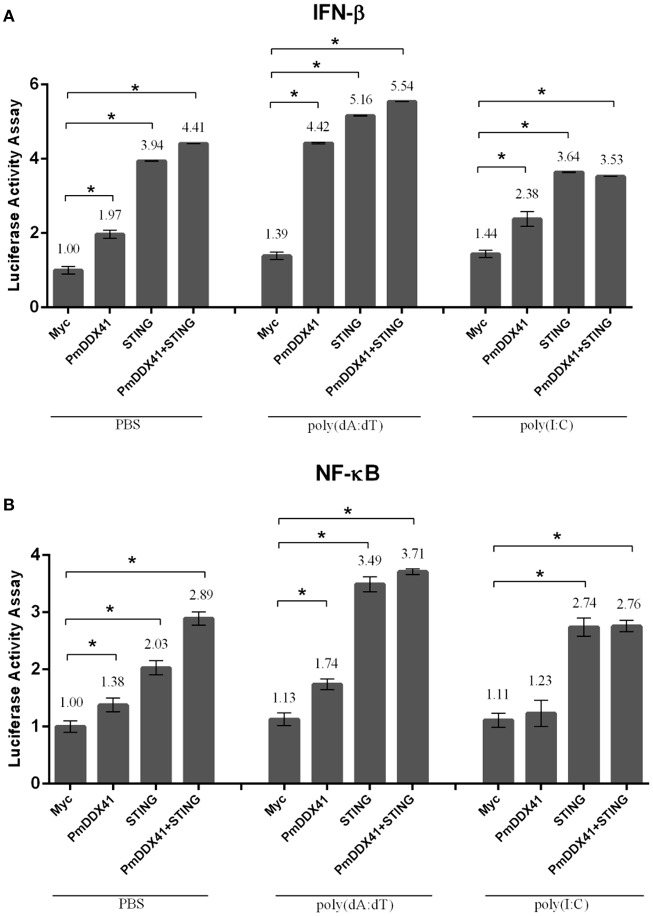
Luciferase assays showing IFN-β **(A)** and NF-κB **(B)** promoter activity in HEK293T cells. Cells were co-transfected with 0.5 μg/well of Flag-tagged-*Pm*DDX41 expression plasmid and 0.5 μg/well of Myc-tagged-*Mm*STING together with IFN-β-Luc and/or NF-κB-Luc (IFN-β-Luc; 0.1 μg/well, NF-κB-Luc; 0.1 μg/well) and the renilla luciferase reporters, pRL-TK (0.01 μg/well). Cells were then stimulated with poly (dA:dT) and HMW poly(I:C). Luciferase assays were performed after 6 h of stimulation. Annotations show significant differences (*p* < 0.05) between normal cells and stimulated cells. The asterisks above each bar indicate mean values that are significantly different (*p* < 0.05).

To further elucidate the involvement of *Pm*DDX41 in antiviral innate immunity, we transfected the expression plasmid for *Pm*DDX41 protein into HEK293T cells with a luciferase reporter plasmid driven by the IFN-β or NF-κB promoter. In normal conditions, overexpression of *Pm*DDX41 significantly induced the luciferase activity of the IFN-β or NF-κB promoters by 1.97- or 1.38-fold, respectively. When cells were stimulated with poly(dA:dT), *Pm*DDX41 enhanced IFN-β and the NF-κB promoter activity by 4.42- and 1.74-fold, respectively (Figures 5A,B). There was no induction of promoter activation following HMW poly(I:C) stimulation compared to control PBS.

To investigate the function of *Pm*DDX41 in the cytosolic nucleic acid sensing pathway, we explored the mediation of *Pm*DDX41 by STING, a molecule which acts as an adaptor for cytosolic nucleic acids in the innate immune response. Since STING is also conserved across different species, we used mouse STING (*Mm*STING) for co-transfection with *Pm*DDX41 in HEK293T cells; this created a cross-species study of the *Pm*DDX41-*Mm*STING-mediated immune signaling pathway. Figures 5A,B show that the overexpression of *Mm*STING in HEK293T cells significantly induced the activation of IFN-β and NF-κB promoters by 3.94- and 2.03-fold, respectively and by 5.16- and 3.49-fold when stimulated with poly(dA:dT), respectively. The co-expression of *Pm*DDX41 and *Mm*STING synergistically activated the activity of IFN-β and NF-κB promoters by 4.41- and 2.89-fold, respectively and by 5.54- and 3.71-fold when stimulated with poly(dA:dT), respectively. In contrast, activation of the IFN-β and NF-κB promoters by the overexpression of *Pm*DDX41, *Mm*STING or *Pm*DDX41 and *Mm*STING was not induced after HMW poly(I:C) stimulation.

### The Interaction of *Pm*DDX41 With *Mm*STING in HEK293T Cells

To further investigate the function of *Pm*DDX41 and *Mm*STING, recombinant of *Pm*DDX41 and *Mm*STING proteins were produced in HEK293T cells. The cells were transfected with Flag-tagged-*Pm*DDX41 or Myc-tagged-*Mm*STING for 24 h. The r*Pm*DDX41 or r*Mm*STING in HEK293T cells were detected by immunoblotting using anti-Flag or anti-Myc antibody, respectively (Supplementary Figures 1A,B).

**Figure 6:**
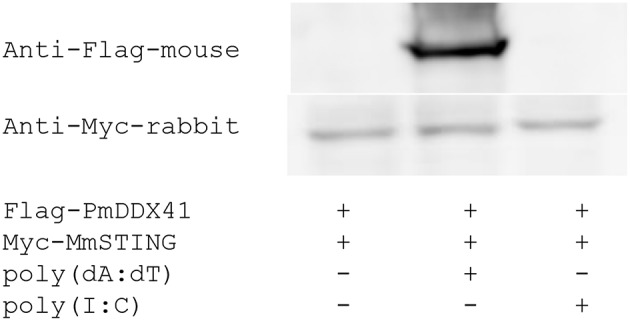
Interaction of *Pm*DDX41 and *Mm*STING in HEK293T cells. HEK293T cells were co-transfected to express the Flag-tagged full-length *Pm*DDX41 and Myc-tagged STING proteins for 24 h. Cells were then stimulated with poly(dA:dT) and HMW poly(I:C) for 6 h. Crude proteins were then extracted and analyzed by immunoblotting with horseradish peroxidase-conjugated anti-Flag and anti-Myc antibodies.

To verify whether *Pm*DDX41 generates a signal by interacting with STING, we performed co-immunoprecipitation (Co-IP). HEK293T cells were co-transfected with Flag-tagged-*Pm*DDX41 and Myc-tagged *Mm*STING. The Flag- or Myc-tagged protein was then detected by Western blotting using an anti-Flag or anti-Myc antibody, respectively. The cell lysates were precipitated with an anti-Myc antibody conjugated with protein A-sepharose beads before being detected by antibody. Our results showed that *Pm*DDX41 could interact with *Mm*STING after poly(dA:dT) stimulation but not HMW poly(I:C) stimulation (Figure 6).

### The Function of *Pm*DDX41 Domains in *Mm*STING-dependent Innate Immune Signaling Pathways

**Figure 7:**
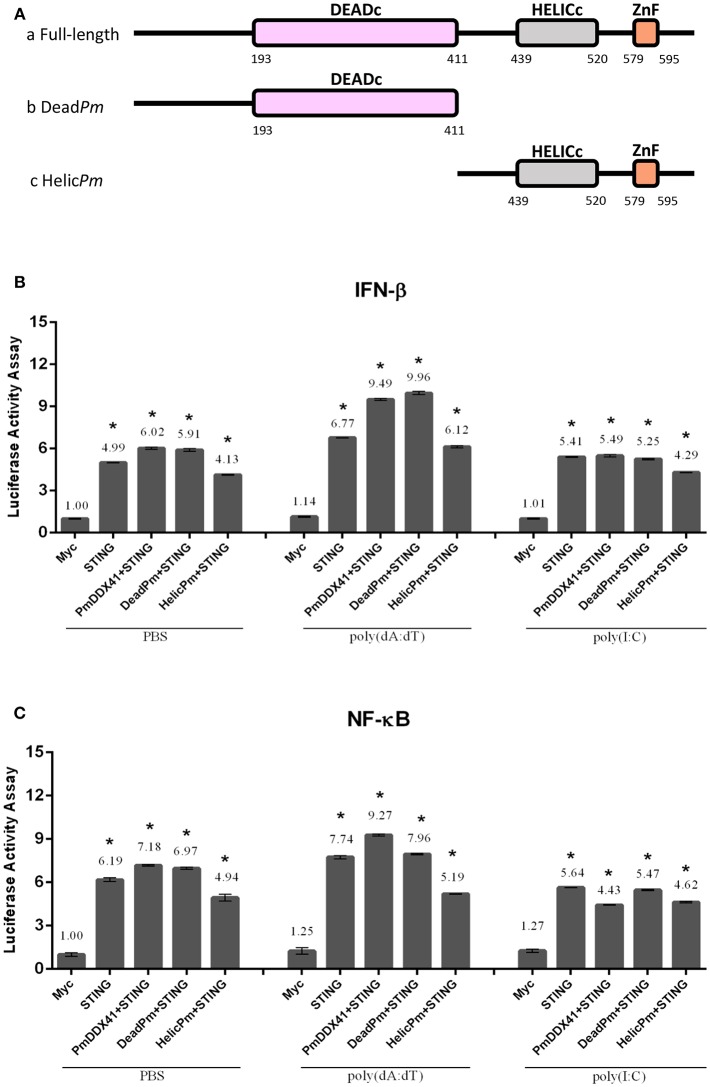
Functional studies of different *Pm*DDX41 domains in the activation of IFN-β and NF-κB signaling pathways. Schematic diagram of full-length and mutated *Pm*DDX41 fragments **(A)**. HEK293T cells were co-transfected with 0.5 μg/well of full-length or mutated *Pm*DDX41 expression vector, or empty vector, and with 0.5 μg/well of Myc-tagged-*Mm*STING together with IFN-β-Luc and/or NF-κB-Luc (IFN-β-Luc; 0.1 μg/well, NF-κB-Luc; 0.1 μg/well) and the renilla luciferase reporter, pRL-TK (0.01 μg/well) and stimulated with poly(dA:dT) or HMW poly(I:C). Luciferase expression of IFN-β **(B)** and NF-κB **(C)** promoters was measured after 6 h of stimulation. Annotations show significant differences (*p* < 0.05) between normal cells and stimulated cells. The asterisks above each bar indicate mean values that are significantly different (*p* < 0.05).

To further investigate the functional roles of different *Pm*DDX41 domains in the DNA sensing signaling pathway, we constructed deletion mutant plasmids lacking the HELICc domain and the DEADc domain (Figure 7A). We then transfected these mutant plasmids into HEK293T cells with a luciferase reporter plasmid driven by the IFN-β or NF-κB promoter. We found that the co-transfection of *Pm*DDX41 or DEADc domain (Dead*Pm*) with *Mm*STING, significantly activated (*p* < 0.01) the promoter activity of IFN-β and NF-κB compared to cells overexpressing *Mm*STING under both stimulated and non-stimulated conditions (Figures 7B,C). There was no significant activation of these reporters in cells co-expressing the HELICc domain and *Mm*STING compared to cells overexpressing *Mm*STING. These results indicated that the DEADc domain of *Pm*DDX41 is required for activation of the *Pm*DDX41–*Mm*STING-mediated IFN-β and NF-κB signaling pathways.

**Figure 8:**
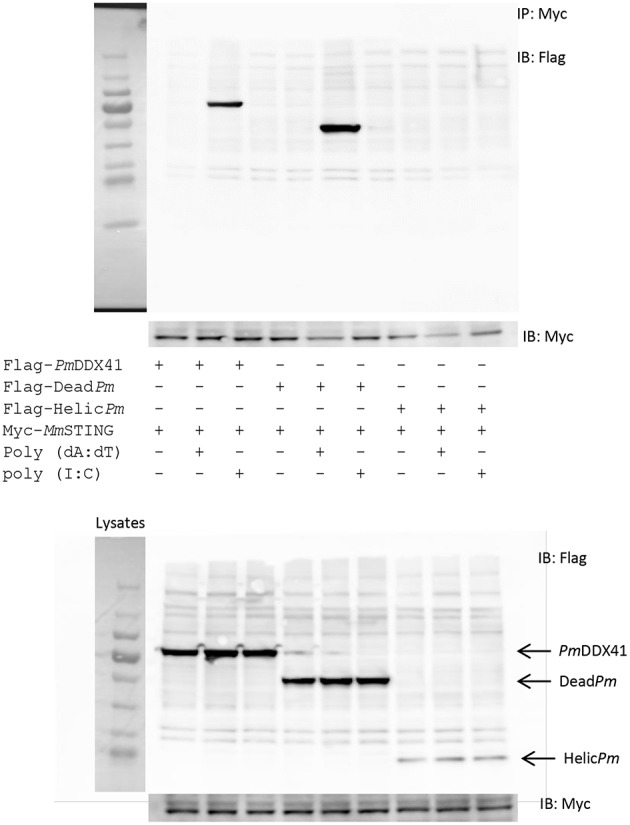
Interactions of different *Pm*DDX41 domains and *Mm*STING in HEK293T cells. Cells were co-transfected with Flag-tagged-mutated-*Pm*DDX41 and Myc-tagged-*Mm*STING for 24 h. Cells were then stimulated with poly(dA:dT) and HMW poly(I:C) for 6 h. Proteins were then extracted. Co-immunoprecipitation assays with anti-Myc antibody (IP: Myc), and Western blotting analyses were then performed with anti-Flag (IB: Flag) or anti-Myc (IB: Myc) antibodies. Expression of the transfected plasmids were analyzed with anti-Flag and anti-Myc antibodies in whole cell lysates.

In order to acquire more supportive evidence, we performed a Co-IP assay in HEK293T cells overexpressing Myc-tagged-STING and Flag-tagged-DDX41 or Flag-tagged-Dead*Pm* or Flag-tagged-HELICc*Pm*. This experiment aimed to evaluate interactions between *Mm*STING and *Pm*DDX41 under normal conditions, and following stimulation by poly(dA:dT) or HMW poly(I:C). We found that *Pm*DDX41 and the DEADc domain could bind only to *Mm*STING after poly(dA:dT) stimulation but not after HMW poly(I:C) stimulation (Figure 8). These results suggest that *Pm*DDX41 binds to *Mm*STING through the DEADc domain and then stimulates the DNA sensing pathway.

**Figure 9:**
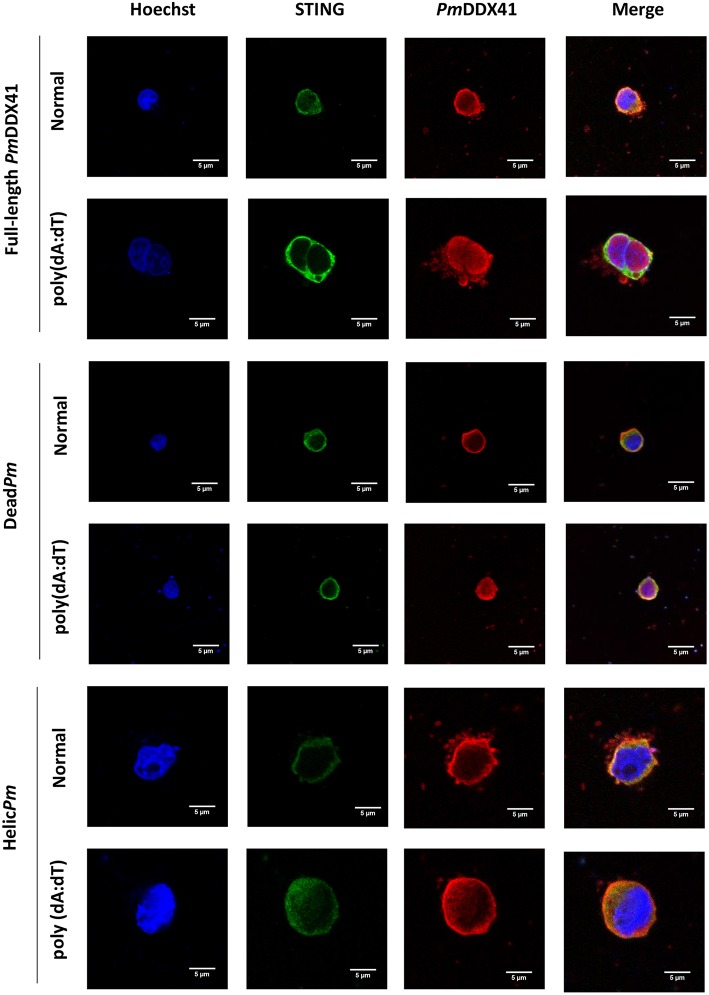
Fluorescence confocal microscopy of mutated-*Pm*DDX41. HEK293T cells were co-transfected with expression plasmids for Myc-tagged STING and Flag-tagged *Pm*DDX41 or Flag-tagged Dead*Pm* or Flag-tagged Helic*Pm*. Cells were then stimulated with 1 μg/ml of poly(dA:dT) for 6 h. HEK293T cells were then stained with an anti-Myc antibody conjugated with Alexa Fluor 488 (green) and an anti-Flag antibody conjugated with Alexa Fluor 568 (red). Nuclei were stained with Hoechst 33342 (blue). Fluorescence images were acquired by laser-scanning confocal microscopy (Zeiss LSM-700; original magnification, 63×).

Immunofluorescence analysis also showed that in normal conditions, *Pm*DDX41, the DEADc domain protein and the HELICc domain protein were localized with *Mm*STING in the cytoplasm. Following stimulation with poly(dA:dT), the expression of *Pm*DDX41 and the DEADc domain were induced and both proteins were found in the cytoplasm and nucleus (Figure 9); *Mm*STING was only located in the cytoplasm. In contrast, the HELICc domain of *Pm*DDX41 remained in the cytoplasm following stimulation (Figure 9).

## Discussion

DDX41 is a member of the DExD/H-box helicase superfamily and acts as an intracellular DNA sensor which triggers the innate immune response against viral infection in mammalian cells (22). *Pm*DDX41 was the first DNA sensor molecule to be identified in the shrimp *P. monodon* and contains three conserved domains; it also shares high similarity with the vertebrates DDX41 (17), thus supporting the fact that DDX41 family members have been highly conserved during the evolution of vertebrates and invertebrates with regards to innate immunity. Suppression of *Pm*DDX41 resulted in a more rapid death of WSSV-infected shrimp and a reduction in the expression of several immune genes. Although DDX41 has been studied extensively in humans, mice, and fish, very few studies have been carried out in invertebrates. In the present study, we further investigated the function of *Pm*DDX41 in innate immune DNA sensing pathways in both shrimp and a human cell line, HEK293T.

The injection of poly(dA:dT) and HMW poly(I:C) into shrimp resulted in the induction of *Pm*DDX41 expression; a higher response was observed in response to poly(dA:dT) stimulation than poly(I:C) stimulation. Our previous study reported that *Pm*DDX41 was up-regulated upon infection with the DNA virus, WSSV, but was down-regulated after being challenged with YHV, an RNA virus (17). These results indicated that *Pm*DDX41 may act as a DNA sensor that responds more specifically to DNA viruses and mimic DNA than RNA viruses or mimic RNA. In the chicken, DDX41 was shown to be up-regulated by dsDNA analog poly(dA:dT) but not by dsRNA or RNA virus (23). DDX41 is known to act as a cytosolic DNA sensor in vertebrates. In the present study, we investigated the localization of *Pm*DDX41 in shrimp hemocytes in non-infected and WSSV-infected shrimp. We found that *Pm*DDX41 was localized mainly in the cytoplasm of non-infected shrimp hemocytes. In contrast, in WSSV-infected shrimp, *Pm*DDX41 was distributed in both the cytoplasm and nucleus. Similar results were observed when HEK293T cells were transfected with *Pm*DDX41 and stimulated with poly(dA:dT) and HMW poly(I:C). HEK293T cells, exhibit a naturally low or defective expression of endogenous DDX41. In our experiments, we used STING as a model system with which to understand the cellular localization of *Pm*DDX41. In duck, DDX41 protein was also found predominantly in the cytoplasm (24). In contrast, zebrafish *Dr*DDX41 was detected in the nucleus of HEK293T cells and was transported from the nucleus into the cytoplasm when cells were stimulated with poly(dA:dT) (9). The DDX41 proteins are very conserved and contain two nuclear localization signals (NLSs), motifs that mediate the transport of nuclear proteins into the nucleus. These NLSs were also found in *Pm*DDX41. Interestingly, the N-terminal 1–194 amino acids of a homolog of DDX41, Abstrakt, from *Drosophila* have been reported to play a role in the translocation of this protein to the nucleus (25). These observations suggested that *Pm*DDX41 is a trafficking protein originally located in the cytoplasm but transported into the nucleus in response to stimulation.

STING, also referred to in previous publications as MPYS, MITA, ERIS, and TRIM173, was discovered using a cDNA expression library designed to isolate molecules that activated the IFN-β promoter (26). Moreover, STING is a central adaptor protein which connects various innate immune signaling pathways by associating with IKKβ or TBK1 to activate the synthesis of type I IFN. In this study, we used STING from the mouse (*Mm*STING) to further elucidate the role of *Pm*DDX41-MmSTING mediated type I IFN and NF-κB induction in HEK293T cells. Our results showed that the IFN-β and NF-κB promoters were activated by *Pm*DDX41. Furthermore, following stimulation with mimic poly(dA:dT), the activities of these promoters were significantly increased, although there was no significant change after stimulation with mimic HMW poly(I:C). Interestingly, the collective overexpression of both *Pm*DDX41 and the membrane-associated adaptor STING had a synergistic effect in promoting IFN-β and NF-κB promoter activity. In the mouse fibroblast cell line L929, the overexpression of DDX41 and STING led to higher activation of the IFN-β promoter (5). To further confirm the interaction between *Pm*DDX41 and *Mm*STING in HEK293T cells, we performed a co-immunoprecipitation assay. This assay clearly showed that *Pm*DDX41 interacted with *Mm*STING after poly(dA:dT) stimulation. Similarly, *Danio rerio Dr*DDX41 was previously shown to bind to *Dr*STING following stimulation with poly (dA:dT) (9). Moreover, we found that *Pm*DDX41 localized together with *Mm*STING in the cytoplasm but following stimulation, some *Pm*DDX41 was translocated into the nucleus while *Mm*STING remained in the cytoplasm. In vertebrates, the DDX41-STING complex was shown to locate in the cytosol and after stimulation with poly(dA:dT), the expression of DDX41 and STING in the endoplasmic reticulum and mitochondria was reduced; however, there was higher levels of DDX41 and STING expression in the endosomes (5). In murine embryonic fibroblasts, STING translocated with TBK1 from the endoplasmic reticulum to the endosome (27). In shrimp, *Pm*DDX41 may exhibit a dual function by sensing DNA in the cytosol and interacting with *Mm*STING to activate the immune signaling pathways and by entering into the nucleus to perform some unknown function. Further studies are now required to elucidate the precise nuclear function of *Pm*DDX41.

In addition, we performed a study to identify the crucial domain responsible for the function of *Pm*DDX41. Based on the predicted domains of *Pm*DDX41, two mutants were constructed to investigate the functional role of these mutants in the activation of IFN-β and NF-κB signaling pathways. The activity of IFN-β and NF-κB promoters were significantly enhanced by co-transfection with the full-length *Pm*DDX41 or the DEADc domain and *Mm*STING. Furthermore, *Pm*DDX41, and its mutant containing the DEADc domain, could interact with *Mm*STING after stimulation with poly (dA:dT). Previous *in vitro* studies using murine dendritic cells showed that DDX41 acts as a cytosolic sensor by binding to synthetic double-stranded DNA (dsDNA) through a DEADc domain (28). These results indicate that the DEADc domain of *Pm*DDX41 is responsible for interacting with *Mm*STING to activate the IFN-β and NF-κB signaling pathways.

Our previous research demonstrated that the knockdown of *Pm*DDX41 caused a reduction in the expression of genes involved in several signal transduction pathways (*Pm*IKKβ, *Pm*IKKε, *Pm*Relish, and *Pm*Dorsal) and antimicrobial peptides (*Pm*PEN3, *Pm*PEN5, and ALF*Pm*6) (17), suggesting that these immune molecules might act downstream of the dsDNA-DDX41-mediated signaling pathways. In shrimp, Toll and IMD signaling pathways regulate the synthesis of several AMPs upon pathogen infection (29). In addition, two IKK homologs, *Lv*IKKβ and *Lv*IKKε, have been identified from Pacific white shrimp these homologs are related to the NF-κB signaling pathway. *Lv*IKKβ also strongly induced NF-κB activity in HEK293T cells, while *Lv*IKKβ, but not *Lv*IKKε, caused the activation of antimicrobial peptides (AMPs), including the Penaeidins. Moreover, the silencing of *Lv*IKKβ or *Lv*IKKε reduced the expression of *L. vannamei* AMPs, including *Lv*PEN2, *Lv*PEN3, *Lv*PEN4, *Lv*lysozyme, *Lv*Crustin1, and *Lv*Crustin2, indicating that the IKK-NF-κB signaling pathway could activate the expression of shrimp AMPs. Recently, a STING ortholog was identified and functionally characterized in shrimp (*Lv*STING) (30) and was found to play a key role in bacterial defense mechanisms (30).

In summary, *Pm*DDX41 is a homolog of human and mouse DDX41 and is highly conserved across a range of vertebrate and invertebrate species. Furthermore, *Pm*DDX41 is likely play a key role in the innate immune response against WSSV infection by binding to the viral DNA via the DEADc domain in the cytosol. *Pm*DDX41 interacts with a STING adaptor in the cytoplasm and initiates a signal by activating downstream regulators, thus leading to the production of antiviral genes, including cytokine-like molecules and AMPs. This research extends our knowledge with regards to the role of *Pm*DDX41 as a cytosolic DNA sensor in the innate immune response to viral infection in the shrimp *P. monodon*.

## Data Availability

The raw data supporting the conclusions of this manuscript will be made available by the authors, without undue reservation, to any qualified researcher.

## Author Contributions

AT and PA contributed to the experimental design and helped to acquire funding. TK helped with the experimental design and provided some resources. SS performed the experiments and wrote the manuscript. AT and TK reviewed, edited, and approved the final version of the manuscript.

### Conflict of Interest Statement

The authors declare that the research was conducted in the absence of any commercial or financial relationships that could be construed as a potential conflict of interest.

## References

[1] Kawai T Akira S. The role of pattern-recognition receptors in innate immunity: update on Toll-like receptors. Nat Immunol. (2010) 11:373–84. 10.1038/ni.1863 20404851

[2] Premecz G Markovits A Foldes I. Signal transducing mechanisms in interferon action (a brief review). Acta Microbiol Hung. (1993) 40:131–40. 7514332

[3] Takeuchi O Akira S. Innate immunity to virus infection. Immunol Rev. (2009) 227:75–86. 10.1111/j.1600-065X.2008.00737.x 19120477 PMC5489343

[4] Cardoso SR Ryan G Walne AJ Ellison A Lowe R Tummala H. Germline heterozygous DDX41 variants in a subset of familial myelodysplasia and acute myeloid leukemia. Leukemia. (2016) 30:2083–6. 10.1038/leu.2016.124 27133828 PMC5008455

[5] Zhang Z Yuan B Bao M Lu N Kim T Liu YJ. The helicase DDX41 senses intracellular DNA mediated by the adaptor STING in dendritic cells. Nat Immunol. (2011) 12:959–65. 10.1038/ni.2091 21892174 PMC3671854

[6] Omura H Oikawa D Nakane T Kato M Ishii R Ishitani R. Structural and Functional Analysis of DDX41: a bispecific immune receptor for DNA and cyclic dinucleotide. Sci Rep. (2016) 6:34756. 10.1038/srep34756 27721487 PMC5056382

[7] Parvatiyar K Zhang Z Teles RM Ouyang S Jiang Y Iyer SS. The helicase DDX41 recognizes the bacterial secondary messengers cyclic di-GMP and cyclic di-AMP to activate a type I interferon immune response. Nat Immunol. (2012) 13:1155–61. 10.1038/ni.2460 23142775 PMC3501571

[8] Chen H Sun H You F Sun W Zhou X Chen L. Activation of STAT6 by STING is critical for antiviral innate immunity. Cell. (2011) 147:436–46. 10.1016/j.cell.2011.09.022 22000020

[9] Ma JX Li JY Fan DD Feng W Lin AF Xiang LX. Identification of DEAD-box RNA helicase DDX41 as a trafficking protein that involves in multiple innate immune signaling pathways in a Zebrafish model. Front Immunol. (2018) 9:1327. 10.3389/fimmu.2018.01327 29942316 PMC6005158

[10] Liu J Huang Y Huang X Li C Ni SW Yu Y. Grouper DDX41 exerts antiviral activity against fish iridovirus and nodavirus infection. Fish Shellfish Immunol. (2019) 91:40–9. 10.1016/j.fsi.2019.05.019 31082519

[11] Quynh NT Hikima J Kim YR Fagutao FF Kim MS Aoki T. The cytosolic sensor, DDX41, activates antiviral and inflammatory immunity in response to stimulation with double-stranded DNA adherent cells of the olive flounder, *Paralichthys olivaceus*. Fish Shellfish Immunol. (2015) 44:576–83. 10.1016/j.fsi.2015.03.008 25776036

[12] Irion U Leptin M. Developmental and cell biological functions of the *Drosophila*. DEAD-box protein abstrakt. Curr Biol. (1999) 9:1373–81. 10607561 10.1016/s0960-9822(00)80082-2

[13] Liu Y Gordesky-Gold B Leney-Greene M Weinbren NL Tudor M Cherry S. Inflammation-induced, STING-dependent autophagy restricts zika virus infection in the drosophila brain. Cell Host Microbe. (2018) 24:57–68.e53. 10.1016/j.chom.2018.05.022 29934091 PMC6173519

[14] Wang PH He JG. Nucleic acid sensing in invertebrate antiviral immunity. Int Rev Cell Mol Biol. (2019) 345:287–360. 10.1016/bs.ircmb.2018.11.002 30904195

[15] Green TJ Speck P. Antiviral defense and innate immune memory in the oyster. Viruses. (2018) 10:133. 10.3390/v10030133 29547519 PMC5869526

[16] Tang X Huang B Zhang L Li L Zhang G. TANK-binding kinase-1 broadly affects oyster immune response to bacteria and viruses. Fish Shellfish Immunol. (2016) 56:330–5. 10.1016/j.fsi.2016.07.011 27422757

[17] Soponpong S Amparyup P Tassanakajon A. A cytosolic sensor, *Pm*DDX41, mediates antiviral immune response in black tiger shrimp *Penaeus monodon*. Dev Comp Immunol. (2018) 81:291–302. 10.1016/j.dci.2017.12.013 29248385

[18] Amparyup P Sutthangkul J Charoensapsri W Tassanakajon A. Pattern recognition protein binds to lipopolysaccharide and beta-1,3-glucan and activates shrimp prophenoloxidase system. J Biol Chem. (2012) 287:10060–9. 10.1074/jbc.M111.294744 22235126 PMC3322982

[19] Pfaffl MW. A new mathematical model for relative quantification in real-time RT-PCR. Nucleic Acids Res. (2001) 29:e45. 10.1093/nar/29.9.e45 11328886 PMC55695

[20] Kawai T Takahashi K Sato S Coban C Kumar H Kato H. IPS-1, an adaptor triggering RIG-I- and Mda5-mediated type I interferon induction. Nat Immunol. (2005) 6:981–8. 10.1038/ni1243 16127453

[21] Tsuchida T Zou J Saitoh T Kumar H Abe T Matsuura Y. The ubiquitin ligase TRIM56 regulates innate immune responses to intracellular double-stranded DNA. Immunity. (2010) 33:765–76. 10.1016/j.immuni.2010.10.013 21074459

[22] Jiang Y Zhu Y Liu ZJ Ouyang S. The emerging roles of the DDX41 protein in immunity and diseases. Protein Cell. (2017) 8:83–9. 10.1007/s13238-016-0303-4 27502187 PMC5291771

[23] Cheng Y Liu Y Wang Y Niu Q Gao Q Fu Q. Chicken DNA virus sensor DDX41 activates IFN-beta signaling pathway dependent on STING. Dev Comp Immunol. (2017) 76:334–42. 10.1016/j.dci.2017.07.001 28684273

[24] Li Y Li H Su N Liu D Luo R Jin H. Molecular cloning and functional characterization of duck DDX41. Dev Comp Immunol. (2018) 88:183–9. 10.1016/j.dci.2018.07.015 30025984

[25] Abdul-Ghani M Hartman KL Ngsee JK. Abstrakt interacts with and regulates the expression of sorting nexin-2. J Cell Physiol. (2005) 204:210–8. 10.1002/jcp.20285 15690390 PMC2963638

[26] Ishikawa H Barber GN. STING is an endoplasmic reticulum adaptor that facilitates innate immune signalling. Nature. (2008) 455:674–8. 10.1038/nature07317 18724357 PMC2804933

[27] Ishikawa H Ma Z Barber GN. STING regulates intracellular DNA-mediated, type I interferon-dependent innate immunity. Nature. (2009) 461:788–92. 10.1038/nature08476 19776740 PMC4664154

[28] Paludan SR Bowie AG. Immune sensing of DNA. Immunity. (2013) 38:870–80. 10.1016/j.immuni.2013.05.004 23706668 PMC3683625

[29] Tassanakajon A Rimphanitchayakit V Visetnan S Amparyup P Somboonwiwat K Charoensapsri W. Shrimp humoral responses against pathogens: antimicrobial peptides and melanization. Dev Comp Immunol. (2018) 80:81–93. 10.1016/j.dci.2017.05.009 28501515

[30] Li H Wang S Lu K Yin B Xiao B Li S. An invertebrate STING from shrimp activates an innate immune defense against bacterial infection. FEBS Lett. (2017) 591:1010–7. 10.1002/1873-3468.12607 28236646

