# Supplementary material for: A Cytosolic Sensor, PmDDX41, Binds Double Stranded-DNA and Triggers the Activation of an Innate Antiviral Response in the Shrimp Penaeus monodon via the STING-Dependent Signaling Pathway
Source: Front Immunol. 2019 Aug 29;10:2069. doi: 10.3389/fimmu.2019.02069 (PMC6736559; doi:10.3389/fimmu.2019.02069)
Supplement: Supplementary file 1 [file Data_Sheet_1.docx]

Supplementary Material

A cytosolic sensor, *Pm*DDX41, binds double stranded-DNA and triggers the activation of an innate antiviral response in the shrimp *Penaeus monodon* via the STING-dependent signaling pathway

Suthinee Soponpong, Piti Amparyup, Taro Kawai, Anchalee Tassanakajon^*^

^*^Correspondence: Professor Dr. Anchalee Tassanakajon: anchalee.k@chula.ac.th


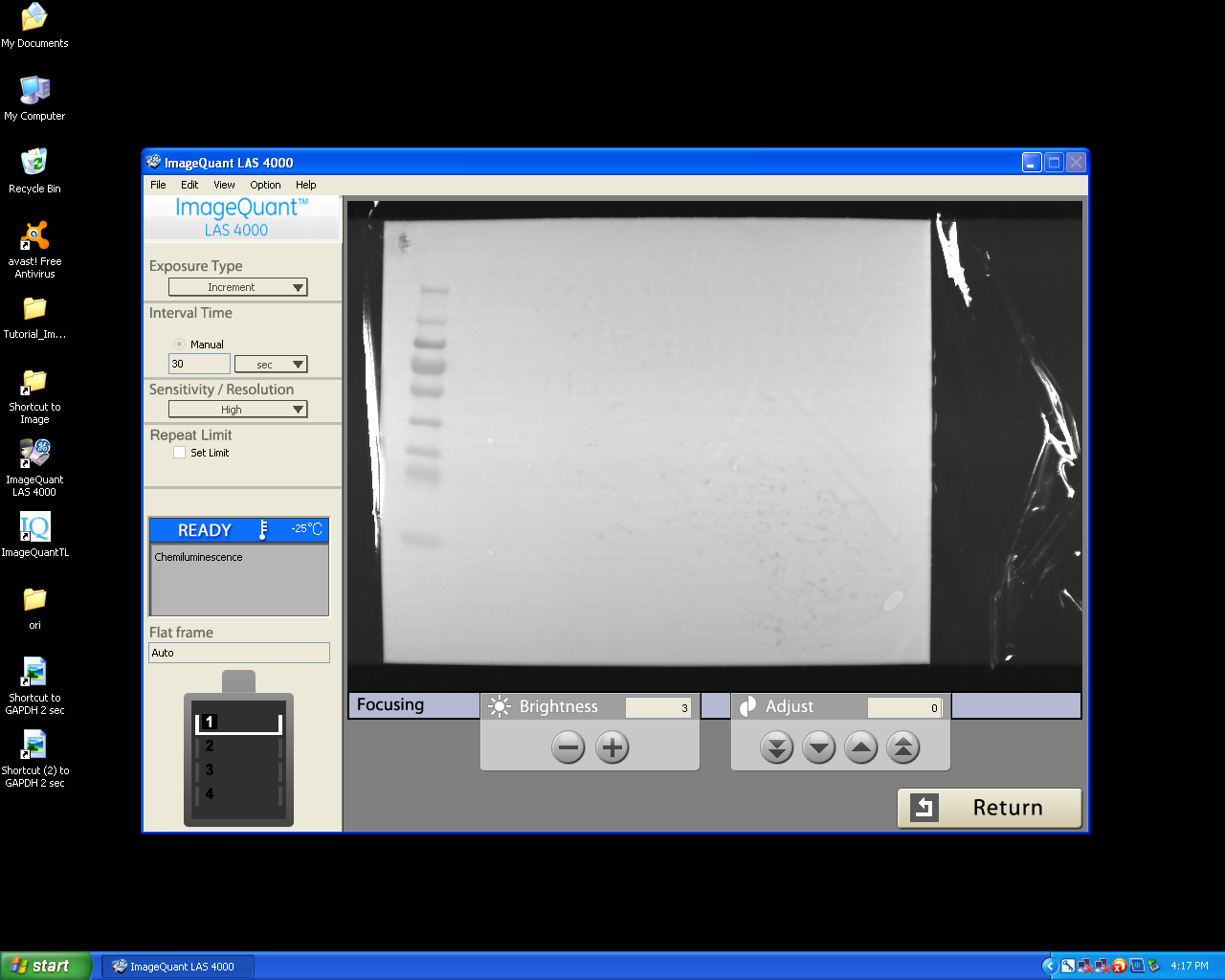

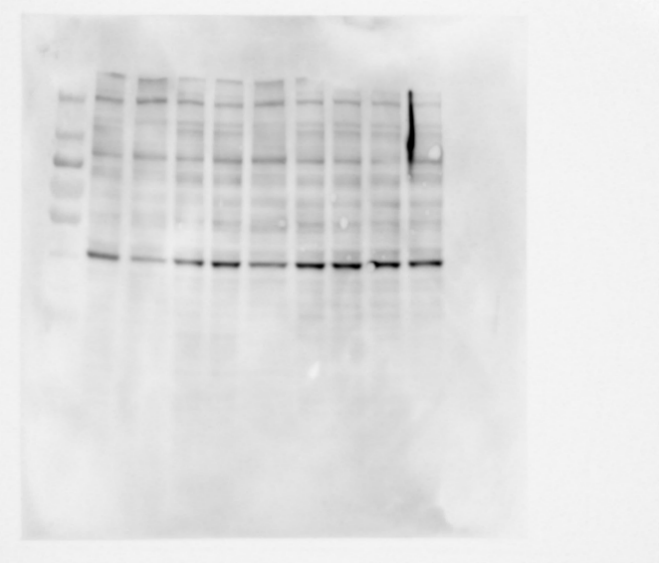


**B**

M

*Mm*STING


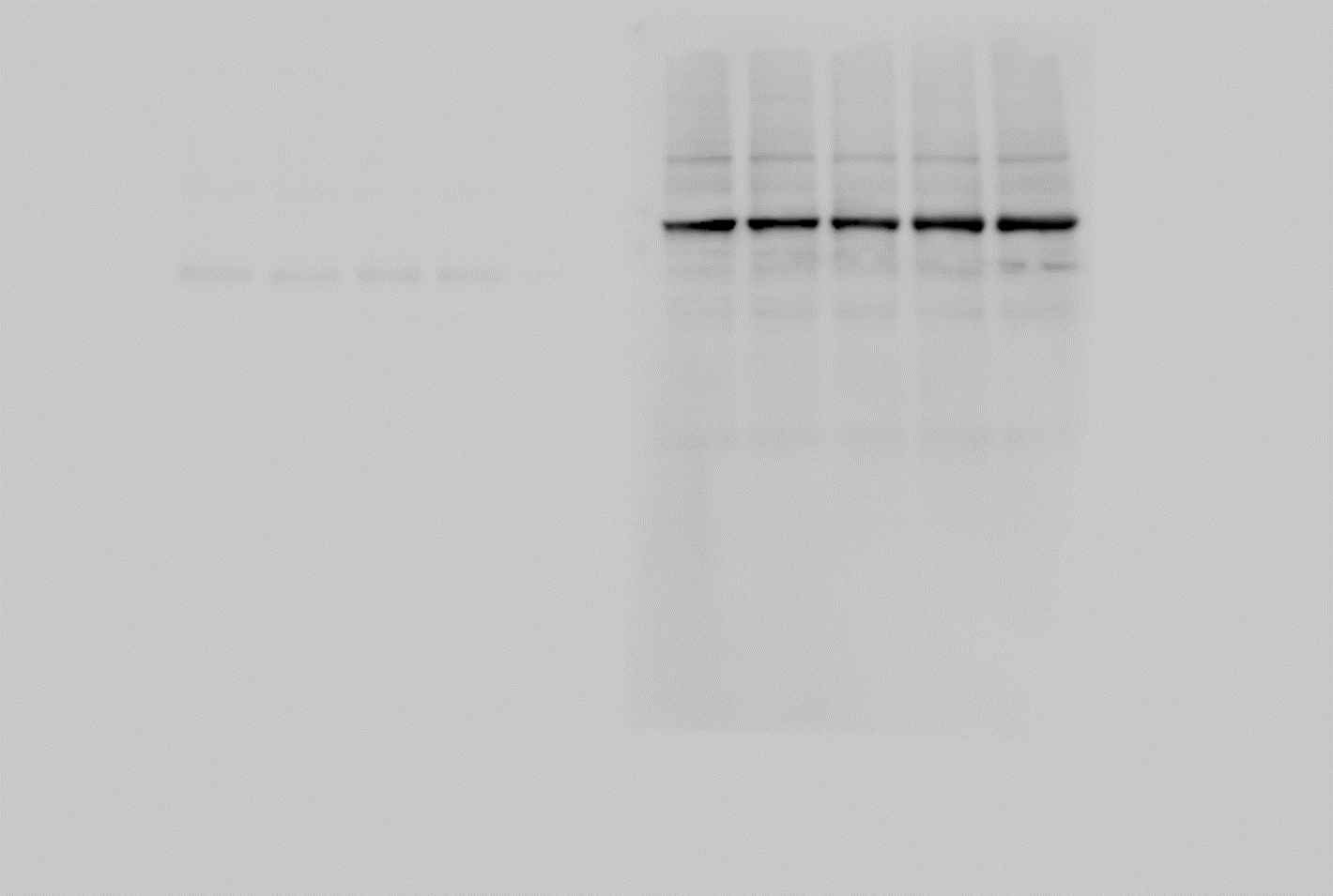

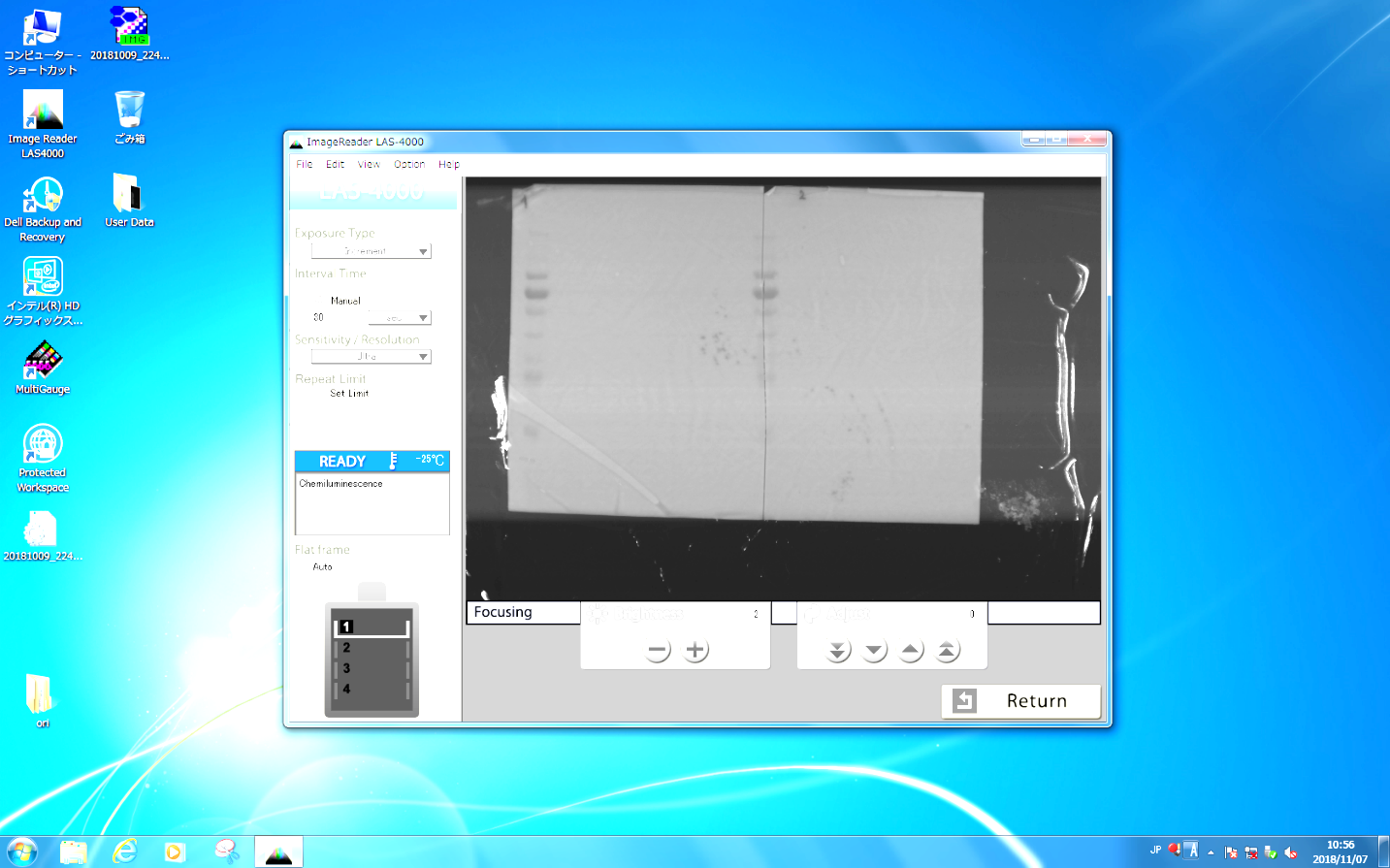


**A**

M

*Pm*DDX41

**Supplement Figure 1.** The western blotting of *Pm*DDX41 and *Mm*STING protein. Overexpression of *Pm*DDX41 (A) and *Mm*STING (B) protein in HEK293T cells which were transfected with 4 µg of recombinant plasmid encoding Flag or Myc tags. After 24h, cells were harvested and sonicated. *Pm*DDX41 and *Mm*STING protein were separated by centrifugation and analyzed by SDS-PAGE. Immunoblotting was carried out with anti–Flag (Sigma), and anti–Myc (Sigma) antibodies. Lane M is the pre-stained protein marker.
